# News: Resources

**Published:** 2011-12

## Environment

### Widely anticipated, climate talks in Durban offer mild progress at best

According to The New York Times, the three days of the Durban climate conference yielded only modest accomplishments. There is now an agreed promise to work toward a new global treaty over the coming years. Also, the agreement has been reached over establishment of a new climate fund. UN’s Secretary General Ban Ki-moon welcomed what he saw as “the climate deal”. However, scientists and critics remained less than impressed with the outcome. They seem unified in their belief that whatever the accomplishments of Durban climate talks, they would surely be insufficient to curb global warming. British newspaper The Guardian tried to see a positive element in the outcome of the meeting, as this agreement has the legally binding character, unlike those that preceded it. The BBC declared EU and the small island states as winners of the talks, while the USA’s manoeuvring space was clearly limited with the upcoming presidential elections. EU representatives argued that the key to success is Chinese adoption of green technology, such as solar panels. Many expressed concerns whether any climate fund would be giving out grants or loans, because the latter could just add to an already unmanageable debt burden in many countries.

### The rules about the new climate fund still not clear

United Nation’s climate summit meeting in Copenhagen two years ago, which was seen by most observers as a fiasco, eventually managed to leave the world with a rare concrete outcome: a pledge, that could be worth up to US$ 100 billion (€77 billion) each year, to assist developing countries to make transition to cleaner energy systems. At the time, Kumi Naidoo, executive director of Greenpeace, has said that the pledge showed that high-income countries were finally taking responsibility for helping low and middle income countries, which contributed very little themselves to climate change. Still, the agreement lacked practical details of where the money should come from and how should it operate to meet its targets. This year, a so-called “Transitional Committee”, which comprises 25 delegates from low and middle income countries and 15 delegates from high-income countries, met four times to try to engineer a fund that should distribute up to US$100 billion (€ 77 billion) annually from the year 2020. Although the committee completed the draft in October, ahead of Durban summit, Saudi Arabia and the United States refused to approve it. Saudi Arabia has been accused for years by environmentalist groups of obstructing climate talks, while the US administration is under intense pressure to limit financing for UN’s climate protection initiatives. The draft was allegedly proposing to give developing countries “direct access” to funds, limiting the role of the World Bank, which is distrusted by many leaders in poor countries; but also to provide donor nations with assurances of “payment for verified results”, potentially allowing them to halt the investments.

### WHO releases global survey of the best and worst cities for air pollution

The World Health Organization released the results of its global survey of the best and worst cities in the world in terms of outdoor air pollution levels. The list relied on country-reported data over the past several years. It was based on the measurement of the levels of airborne particles that are smaller than 10 µm (“so-called PM10s”). The list included almost 1100 cities from all over the world. Apparently, cities in Iran, India, Pakistan and the capital of Mongolia ranked among the worst in the world for air pollution. The cities in the United States and Canada are generally among the best. In this first global survey, only 11 of 91 countries met WHO standards on air quality.

### Climate change alone cannot explain increasing cholera outbreaks

In summer of 2011, New York Times reported that cholera outbreaks seem to be on the increase. However, it quoted a new study from the Tufts University researchers, published in *The American Journal of Tropical Medicine and Hygiene*. The study has found that those outbreaks cannot be explained by global warming alone, and that a much more important factor may be the cycle of droughts and floods along big rivers.

### UN tested warning system for tsunami in North Atlantic and Mediterranean region

A release from the UN said that United Nations-backed North Atlantic and Mediterranean tsunami warning system has passed a first test of its communication network. Major disasters in Indonesia and Japan in recent years, which followed strong earthquakes occurring at the ocean’s bottom, have revealed the horrific power of tsunamis to cause massive mortality and economic damage for human populations. The successful test will be used as a model for the establishment of other regional tsunami warning centres, which should prevent cost on human lives.

## Demography

### World population reaches 7 billion at the end of 2011

It took only 12 years for human population to increase by another billion. According to “The State of World Population 2011”, a report released the UN Population Fund, the number of living humans will reach 7 billion at the end of October 2011. This comes as a continuation of positive demographic trends since the World War II, during which average life expectancy rose from about 48 years (in the early 1950s) to 68 years (in 2010). It is projected that 90% of future population growth will occur in the least developed countries, which will lead to intensified competing for very restricted resources in those countries, poverty and reduced access to health services. The International Conference on Family Planning will be held at the end of 2011 in Dakar, Senegal. It is expected to bring together high-level leaders from Africa and Europe, world-class researchers, and advocates. They will discuss how to access family planning, especially for 215 million women who want to avoid or delay pregnancy, but do not have access to contraception or cannot use it for different reasons.

### An economic exodus from Europe has surely started

Only years ago, the European Union was still considered a premium global sanctuary for all those trying to escape poverty, war and injustice in many other parts of the world. But the recent figures are beginning to show the unthinkable: a growing number of Europeans are leaving the continent and heading south – to Australia and South America. It seems that tens of thousands of Portuguese, Greek and Irish people have already left their homelands this year, with the same about to happen, or already happening, in Spain and Italy. The most surprising are the new migration routes – from Portugal to Angola, Ireland to Australia, Spain to Argentina. This year alone, about 2500 Greek citizens have moved to Australia, but further 40 000 have also “expressed interest” in moving. In Ireland, things are even more dramatic – up to 50 000 people will leave this year, mostly for Australia and the USA. But perhaps most surprisingly, at least 10 000 people have left Portugal, which has life expectancy of about 79 years, for oil-rich Angola, where people live 30 years shorter on the average. Up to 100 000 Portuguese citizens now live in Angola, which is double the number in 2005. Brazilian government also reported that the number of foreigners legally living in Brazil rose from about 1 million a year ago to 1.5 million at the end of 2011, with the number of Portuguese alone rising from about 275 000 to about 325 000 within a year.

### A report issued by the UN tells member states to legalize abortion

The Guardian wrote recently about a “hard-hitting report from the UN special rapporteur on health as a human right”, who said that “…all states must provide safe abortion and contraception for women”. However, there are member states of the UN general assembly that still prosecute women seeking abortion. This report told them very directly that they are infringing woman's human rights. Mr Arnand Grover stated in his report that “…all states should provide safe and legal abortion services for woman – as well as contraception”. In countries that harbor up to 25% of the world's total population, however, it is a criminal offence for a woman to end her pregnancy, unless she had been raped, the pregnancy had been a consequence of an incestuous intercourse, or where her life was at risk. Mr Grover based his report on a rational analysis of the impact of restrictive laws on women's human rights. He also argued that the member states are wrong to prosecute women for illegal drug use or drinking during pregnancy, because “…criminalisation only succeeds in driving women away from the help they need”.

### Get sterilized and drive away in a Nano car

Get sterilized and drive away in a Nano car! Nano is the low cost (US$ 2700, € 2000,) car launched by Tata Motors few years ago. The idea piloted by the health authorities in Rajasthan's Jhunjhunu district in India to check population growth has been adopted by other districts. Apart from the bumper prize, this limited period offer also included motorcycles, TV sets and mixer-grinders to encourage sterilization in the district. The scheme has caught the imagination of the local population and the number of persons opting for sterilization has grown 5-fold compared to the previous year. Rajasthan witnessed a substantial drop in those opting for sterilization in the last decade, but now the authorities in the district are upbeat that they will once again be able to reduce the population growth rate.

### Third child could now land you in prison in Kerala

This will become a reality if the Kerala Women's Code Bill 2011, which is presently being considered by the state government, is implemented. The bill recommends that a fine of INR 10** **000 (US$ 180, € 145), which could be exchanged for three months of imprisonment, is a deserving punishment to any expectant father of a third child in this Indian state. The recommendation is part of the measures intended to encourage population planning for well-being and children's development. Those parents who violate the norms would also be regarded as “legally disqualified persons„. The bill also proposes an incentive for women who marry after the age of 19 and have their first child after they turn 20 years.

## Economy

### High-income countries worried over debts, but poverty keeps shrinking world-wide

As the media in industrialized countries spent much of the year covering Europe's roller-coaster efforts to save the common currency and assist the struggling members – resulting in unexpected withdrawals of Greek and Italian Prime Ministers George Papandreou and Silvio Berlusconi in the process – in the rest of the world the year has not been that concerning in terms of economy. All indicators are now showing that poverty could be shrinking globally. Eleven years ago, the United Nations challenged the world to halve extreme poverty by 2015 as one of the Millennium Development Goals. At this point in time, it is beginning to seem that developing nations may reach that goal. The World Bank's recent estimates projected that the low and middle income economies have, in fact, already reached 80% of that target. The number of people living on less than US$ 1.25 (€ 1) a day is projected to fall to 883 million in 2015, compared with 1.4 billion in 2005 and 1.8 billion in 1990, according World Bank statistics – a decrease in absolute terms which happens against the background of the growing world's population. The outlook for developing countries to reduce hunger, enrol children in primary school, and reach a number of related UN-set benchmarks seems similarly good.

### Critics of G20 summit in Cannes focus on failure to regulate irresponsible lenders

A number of advocacy groups issued a mixed bag of critical and partially praising statements following the most recent G20 summit in Cannes, France. The group ONE, represented by Michael Elliott, its president and chief executive, highlighted the 75 minutes spent on talks about innovative finance for development (the “Robin Hood” or Tobin tax) as the greatest success, along with the speech from Bill Gates and the resulting fact that development has now become a part of the G20s agenda. The UN’s World Food Programme, was “pleased by the G20's decision to exempt food aid from export restrictions and extra taxation”, because “…the move would ensure food assistance continues to reach people affected by hunger as a result of high prices and humanitarian crises”, as WFP’s executive director Josette Sheeran told The Guardian. Some of the most critical tones came from the Jubilee Debt Campaign, which called for debt cancellation rather than loans to bail out private creditors. Tim Jones, policy officer at Jubilee, said for The Guardian: “It is incredible that the in the midst of another global debt crisis, the most powerful countries are still failing to regulate irresponsible lenders. An orderly system is needed to cancel unjust debts, neutral of both creditors and debtors. Yet the G20 seem happy to continue with the debt debacle currently being played out in Europe, as has been seen across the world for the last 30 years. The continuing history of debt crises – from Africa, Latin America, East Asia and now Europe – is that loans to bail out private creditors and enforced austerity do not work. Instead, reckless lenders need to be made responsible for their actions and debts cancelled. Global rules are needed to enable this to happen”.

### Europe’s debt crisis could affect global vaccination funding

The International Finance Facility for Immunisation (IFFIm) has been set up to rapidly accelerate the availability and predictability of funding available for global immunization efforts. The resources raised by IFFIm fund the GAVI Alliance, a public-private partnership that provides funds to purchase and deliver life-saving vaccines and strengthen health services in low-income countries that could not achieve this themselves. The role of IFFIm in this process is to use pledges from donor governments at the GAVI fundraising meetings to sell bonds in the capital markets. The sales of those government bonds then generate instant cash required for GAVI programmes. Now, after Standard & Poor have already put the 15 Euro-zone countries on negative credit watch, they also announced that the IFFIm’s triple-”A” rating would also be downgraded, because France and the Netherlands together account for about a third of the contributions to the IFFIm expected over the next two decades. The bond sales contribute about US$ 1.5 billion (€ 1.2 billion) each year to GAVI programmes, and if the bonds are downgraded, this would increase the overall cost of GAVI programmes – i.e., GAVI would be able to supply less vaccines with the same amount of support, at the expense of higher interest of borrowing, as a result of Standard & Poor’s ratings change.

### Economic improvements could be achieved through collecting the evaded tax

The Guardian's journalist Richard Murphy recently reported on the Tax Justice Network's publication of the research which he has undertaken on its behalf. Using data sourced from the World Bank, CIA World Factbook, the Heritage Foundation and World Health Organisation, he estimated tax evasion for 145 countries in the world, which cover 98% of world's GDP. His estimates indicate that about US$ 3.1 trillion (€ 2.4 trillion) is being lost each year through illegal tax evasion. That is more than 5% of their collective GDP, i.e. more than a half of the money that all those countries combined spend on health care. Mr Murphy makes a point that, across the world, governments pay too little attention to tax evasion, claiming that the issue is smaller than it really is. They have done so because they “mainly look at errors in the tax returns they receive, ignoring the fact that serious tax evaders are outside the system”. He estimates that, had the level of tax evasion in Italy or Greece over the past decade been similar to the level in the UK, both countries would not have had nearly as much external debt as they do now. He concluded that “…if tax evasion had been taken seriously and been tackled in these countries, we would not have a crisis in the Euro-zone today”.

### UK urged to legally prevent vulture funds from preying on world's poorest people

According to The Guardian, the UK is being urged to help close down a legal loophole that lets “vulture funds” use courts in Jersey to claim hundreds of millions of pounds from the world's most unfortunate countries. Max Lawson, head of policy at Oxfam, said that “…the government could close this loophole tomorrow if it wanted to and stop tax havens becoming the 'go-to' destinations for vulture speculators. Vulture funds legally buy up worthless debt when countries are at war, or suffering from a natural disaster, and defaulting on their sovereign debt. Once the country has begun to stabilise, vulture funds cash in their cheap debt deeds, at massively inflated cost to the countries”. In the case to be decided next month before the Jersey court, FG Hemisphere – run by vulture financier Peter Grossman – is trying to collect US$ 100 million (€ 77 million) from the DR Congo on a debt that was originally US$ 3.3 million (€ 2.5 million) and was owed to the former Yugoslav government to build power lines.

## Energy

### Global energy consumption undergoing very strong growth

BP oil and gas company recently released their highly respected annual Statistical Review of World Energy for 2011. Most of the report focused on the very strong growth in global energy consumption. Overall energy consumption growth was 5.6% globally, which was the highest rate since 1973. Oil prices averaged the second highest level ever, with growth rate of 3.1%. The largest oil producers in the world are now Russia (12.9%), Saudi Arabia (12.0%) and the United States (8.7% of total global oil production). Growth in natural gas was 7.4%, the largest in more than 25 years. Coal consumption increased by 7.6%, which is again the highest growth rate since 2003. Coal’s share in global energy consumption rose to 29.6%, with China consuming nearly half of the world’s coal (48.2%). The USA had 24.7% of total global renewable energy consumption, but China had the highest growth rate of renewable energy among large countries (increase of 74.5%). Biofuels production increased by 13.8%, and renewable energy for power generation by 15.5%, the latter led by wind power - which increased by 22.7%. Hydropower consumption grew by 5.3%, with China being the top global producer of hydropower (21% of the global total) and followed by Brazil (11.6%), Canada (10.7%), and the US (7.6%). Finally, global use of nuclear energy grew by 2%.

### Nuclear power development likely to slow down after Fukushima Daiichi nuclear disaster

The tsunami that struck Japan in March 11 left many horrific consequences, but global press was quick to focus on the problems at the flooded Fukushima Daiichi nuclear plant. Although it remained somewhat unclear what the true risks were as the events were unravelling, the media coverage surely made it look like the worst nuclear crisis since Chernobyl. In the aftermath of this tragedy, intense debates flooded the press over whether nuclear power could ever be risk-free. A number of countries decided that the potential consequences of a significant accident were just too big to accept, and they reversed their plans for investing in nuclear plants - in some cases, even shutting down existing plants. The incident will likely slow the global development of nuclear power. The Chernobyl accident had the same effect in the late 80s and early 90s.

### Prices of solar panels halved in 2011

The prices of solar panels decreased rather dramatically in 2011, reaching the levels to only about 50% of the prices typically present in 2010. This is certainly good news for many consumers interested in developing a personal power source for their households. However, many manufacturers of solar panels that were based in the western countries, and were only recently seen as potentially good long-term investments, are now under intense pressure because they can hardly meet their operation costs. Some among them, like Solyndra, were forced to declare bankruptcy in 2011.

### US refiners’ export greater than import for the first time since 1949

This year, the United States’ refiners have exported more finished products than the country imported for the first time since 1949. These products include jet fuel, heating oil and gasoline. Interestingly, this development also highlighted the energy illiteracy among some journalists who reported incorrectly: “…the US had become a net exporter of oil”. This was not even remotely true, because the US is still highly dependent on its oil imports. The difference this year was that the country has been using more of that oil to make finished products, which were then exported back into global markets.

### A temporary victory for environmentalist in the Keystone Pipeline deadlock

Environmentalists groups and lobbies declared a victory after the US Administration announced a delay in approving the controversial Keystone Pipeline. This pipeline was proposed to deliver oil from Canada’s Athabasca oil sands to the USA. Still, this delay may not mean the abandonment of the plans and it will remain interesting to follow further developments.

## Peace & Human Rights

### Apparent decline of war: a short-term historic trend, or a beginning of new era?

Recently, a number of analysts and scholars began to point out that since the late 1940s annual world battle deaths have fallen by more than 90%. It seems that it is not only classical big army clashes that are in decline, but also everything from low-intensity militia conflicts to civil wars. Even terrorism seems to be becoming less frequent and deadly. “If war is really obsolete, it would be one of the most important developments in the history of the human race”, said John Mueller, chair of national security studies at Ohio State University in Columbus and an expert in conflict trends. A similar sentiment is proposed by Joshua S Goldstein, writing for Foreign Policy magazine. He said that that in the past decade there have been fewer wars and fewer deaths in war than at any time in the last century, and hopes that the end of American dominance will not change that. Some of the proposed explanations are that large armies are becoming harder to raise and sustain, technological advance has increased the accuracy of munitions and reduced the amount of collateral damage, and states are finding peaceful ways of resolving their disputes rather than resorting to force. Some fear, though, that the rise of China will inevitably bring it into conflict with the United States. However, Chinese military expenditure, albeit growing fast, is still a distant second with US$ 100 billion (€ 77 billion) a year in comparison to United States' US$ 700 billion (€ 540 billion). Also, Chinese interests are closely linked with those of America, given that its economy depends on exports and that it owns a large amount of US government debt – making a possible conflict entirely counterproductive to both sides. The end of American hegemony will surely be replaced by a multipolar world, in which there will be several very strong actors who may persuade each other that treaties and agreements are a better way than wars.

### Nobel Peace Prize goes to three remarkable ladies for their women's rights work

This year’s Nobel Peace Prize has been awarded to three remarkable ladies: Ellen Johnson Sirleaf, Leymah Gbowee and Tawakkul Karman, for their work in advancing women's rights and the role of women in peace-building efforts. The two are from Liberia and one is a Yemeni, and they were recognized by the Nobel committee for their “non-violent struggle for the safety of women and for women’s rights to full participation in peace-building work”. The committee added that the world “cannot achieve democracy and lasting peace “unless women obtain the same opportunities as men to influence developments at all levels of society”. Johnson Sirleaf, a Harvard-trained economist, was elected president of Liberia in 2005 – Africa's first democratically elected female president. Leymah Gbowee is a trained social worker known for her work on peace-building, truth and reconciliation in Liberia and efforts to advance women's rights across Africa. Tawakkul Karman is a young activist and chair of Women Journalists Without Chains, who has been working to promote human rights in Yemen for years; her arrest helped kick off protests by hundreds of thousands demanding the ouster of President Ali Abdullah Saleh and the creation of a democratic government.

### What will replace the fallen regimes of Northern Africa?

For the first time in decades, the possibility exists that democracy may get introduced to the Middle East. However, it is still very unsure what will replace the fallen regimes of Tunisia, Egypt, and Libya, now that they have ousted autocratic leaders. It is also unclear how will the continuing turmoil in Syria, Yemen, and Bahrain end. However, the era of autocrats – many of whom have been kept in power by a strong backing from the western powers – may indeed be ending. A wind of change has swept the Middle East and Northern Africa, with dictatorial regimes being ousted by pro-democracy protesters, who mainly sought to end tyrannical rule and institutionalized corruption. The demands of the protesters, who were up against a real military might, demanded a new political dispensation, major economic and political reforms and constitutions that will guarantee free speech, dissent, and participatory democracy.

### Europe to focus on democracy and human rights in aid projects

According to The Guardian, the EU will soon adopt its new development policy, in which a far greater focus will be placed on democracy, human rights and governance in its aid programmes. Andris Piebalgs, the EU commissioner for development, unveiled the EU's “agenda for change” and said that human rights and democracy were “…guarantors that economic development was sustainable”. Even with economic worries of its own, the EU is still the world's biggest donor of official development. Mr Piebalgs said that it will seek to promote democratic rights through “good governance and development contracts” set up between the EU and countries receiving support.

### An analysis shows co-occurrence between climate cycles and civil wars

In August, the Guardian wrote about an analysis of 250 conflicts between 1950 and 2004. It implied that 50 of them were “triggered by El Nino”, which translates to “doubling the risk of civil wars”. Researchers believed that the climate phenomenon, known as El Nino, may contribute to unrest by bringing about its hot and dry conditions to tropical nations. The resulting cuts in food production are thought to lead to outbreaks of dissatisfaction, which soon focus on the flaws of regional leaders and are followed by violence. The researchers quote examples of similar cycles in countries from southern Sudan to Indonesia and Peru.

## Food, Water and Sanitation

### Incoming FAO chief highlights three steps to hunger reduction success

The incoming Director General of FAO, Jose Graziano da Silva, recently highlighted three elements that are required within a successful strategy to fight global hunger. He first highlighted the need for political commitment to eradicating hunger in the low-income settings. Second, he suggested involvement and partnership with FAO, WFP and IFAD. Finally, he called for absolute goals beyond the Millennium Development Goals. Talking about Brazil’s experience, Mr Graziano da Silva said that whatever was invested in fight against hunger was rapidly recovered, through extraordinary returns. He stressed that the consumption cycle immediately brought back revenue in taxes and the expenditure lead to new jobs and incomes.

### Millennium Development Goal on drinking water “nearly achieved”

UNICEF and the WHO released a report on the progress being made on Millennium Development Goal (MDG) of improving access to safe drinking water. In view of the experts from these two organizations and their collaborators, this MDG is increasingly likely to be achieved – possibly even ahead of the 2015 target date. The report – entitled “Drinking Water Equity, Safety and Sustainability” – reported that between 1990 and 2008 the proportion of the world's population with access to improved drinking water sources has been increasing from the initial 77% to recent 87%. The MDG target was to halve the proportion of people without sustainable access to safe drinking water, from the 23% down to 12%. We should expect that over the next couple of years this goal will be successfully reached.

### Several food crises looming in Africa, Sahel tops the urgency list

Several food crises are looming in Africa: in Zimbabwe, the Sahel and the Horn of Africa. The situation in Somalia could reportedly worsen if the ban imposed by Al-Shabaab insurgents on 16 aid agencies does not get reversed. And in Kenya, which has also been affected by the drought, followed by recent floods, the crisis may also continue. Africa’s Sahel region is expected to face a severe food shortage over the coming months because of rather erratic rainfall and localized dry spells. Both the EU, UNICEF and UN officials recently warned about this particular crisis. It is also estimated that about seven million people are already facing food shortages in Niger, Chad, Mali, Mauritania, Nigeria and Burkina Faso.

### Current progress rate would take '200 years' to reach UN’s targets on sanitation

According to The Independent, the UN’s Millennium Development Goal to halve the number of people without access to sanitation by 2015 – also known as “target 7C” – has been failing so dramatically, that some of the world's poorest countries would require another 200 years to reach it under the current rate of progress. Nearly a billion people worldwide are presumed to live without access to clean water, while more than two and a half billion live without adequate sanitation. The latter figure includes more than a third of all humans. More than 90% of people who don’t have basic access to sanitation facilities live in only about 30 countries, with the highest absolute numbers in India and China. However, the top ten recipients of water, sanitation and hygiene aid over the past decade have not been those in greatest need, but rather middle- (or even upper-middle-) income countries. Barbara Frost, chief executive of WaterAid, said for The Independent that “…historical and strategic interests still influence where aid is going, rather than the countries and communities where poverty and need is highest” and that “…over the past decade, least developed countries have received only 30 per cent of aid for water, sanitation and hygiene”.

### World Food Program awards Bill Gates for focus on small farmers in poor countries

World Food Program USA’s George McGovern Leadership Award was given to Mr Bill Gates, co-chair of the Bill and Melinda Gates Foundation, for his foundation’s efforts to help small farmers in the developing world overcome hunger and poverty. Mr Gates said that the combination of famine in the Horn of Africa, rising food prices, and a growing population make it critical at this point in time to help poor farmers grow and sell more food. At the G20 Summit in Cannes, France, Mr Gates delivered a report outlining how innovations and partnerships in both health and agriculture can help increase global stability and assist the poorest countries in achieving economic growth and equality.

## Science & Technology

### Apple co-founder Steve Jobs died in California at the age of 56

An inspiration to many of us – an ultimate visionary, the man who managed to truly change the way we spend our time, to improve our world and to develop its most valuable company – Steve Jobs, billionaire co-founder of Apple, has died in California at the age of 56. He was a genius who managed to revolutionize personal computing, mobile telephony, and even music that we listen to. Earlier this year, he stepped down as chief executive of the Apple, which he helped set up in 1976, citing illness – he had been battling an unusual form of pancreatic cancer. Apple released a statement in which they paid tribute to their leader: “Steve's brilliance, passion and energy were the source of countless innovations that enrich and improve all of our lives … The world is immeasurably better because of Steve”. Mr Jobs was one of the pioneers of Silicon Valley, who did a lot to help establish this region as the global technology hub. He founded Apple with Mr Steve Wozniak, his childhood friend, and they marketed together the world's first personal computer, the Apple II. He had to leave Apple after a dramatic and uneasy boardroom battle in 1985, a move for which Mr Jobs later claimed was the best thing that could have happened to him. He went on to buy Pixar, the company which now keeps creating the biggest animated movies of our time. However, after 11 years he returned to Apple when it was being written off by rivals, and engineered possibly the most remarkable comeback in business history. Apple was briefly the most valuable company in the world earlier this year, producing US$ 65.2 billion (€ 50.3 billion) a year in revenue, compared with only US$ 7.1 billion (€ 5.4 billion) in 1997. Mr Jobs' inventions that will remain remembered include Apple I (1976), a computer for hobbyists and engineers; Apple II (1977), one of the first successful personal computers; Lisa (1983), the first commercial computer with a graphical user interface, with icons, windows and a cursor controlled by a mouse; Macintosh (1984), an improved version of Lisa, coupled with a laser printer; NeXT computer (1989), a powerful workstation computer on which the world's first web browser was created; iMac (1998), strikingly designed as a bubble of blue plastic that enclosed both the monitor and the computer; iPod (2001), the first successful digital music player with a hard drive; iTunes store (2003), which simplified buying digital music and became the largest music retailer in the US in 2008; iPhone (2007), which did for the phone experience what the Macintosh did for personal computing; and iPad (2010), the first successful tablet computer. To date, more than a million people from across the world have shared their memories, thoughts, and feelings about Steve Jobs on Apple's Web site. One thing they all have in common how they’ve been touched by Mr Jobs' passion and creativity.

### The first report of substantial effectiveness for malaria vaccine

This year has seen the first report of substantial effectiveness for a vaccine against a parasitic disease in humans. The New England Journal of Medicine published an interim report of RTS,S Clinical Trials Partnership's large multicenter phase 3 trial of RTS,S/AS01 Plasmodium falciparum malaria vaccine. The trial involved more than 15 000 children in two age categories — 6-12 weeks and 5-17 months. The study described vaccine efficacy against P. falciparum malaria in the first 6000 children in the older age category with an evaluation of the first 250 cases of severe malaria from both age groups. The vaccine has been developed through a public–private partnership between GlaxoSmithKline and the Program for Appropriate Technology in Health (PATH) Malaria Vaccine Initiative, supported by the Bill and Melinda Gates Foundation. The vaccine is a hybrid construct of the hepatitis B surface antigen fused with a recombinant antigen derived from part of the circumsporozoite protein, the protein coat of the sporozoite. Sporozoite is inoculated by the feeding anopheline mosquito and it invades liver cells, where it multiplies before entering blood. The vaccine is intended for use among infants and young children in sub-Saharan Africa. The interim results showed 55% reduction rate against all malaria episodes and 35% reduction of severe malaria. Although most scientists hailed this study as a massive breakthrough and a major milestone achieved in global health research, those more sceptical pointed out that it not usual practice to publish the results of trials in pieces, and there does not seem to be a clear scientific reason why this trial has been reported with less than half the efficacy results available. The RTS,S/AS01 P. falciparum malaria vaccine should become available in about 3 years and the World Health Organization (WHO) indicated that it could recommend it for use in some African countries as early as 2015, depending on the full phase 3 trial results which are expected in 2014.

### Study shows that HIV infection can be prevented with antiretroviral therapy

HIV–AIDS pandemic started 30 years ago and resulted in 60 million infections and 30 million deaths to date. However, the introduction of potent combination antiretroviral treatment in 1996 and the public health approach to treatment in poor settings in 2002 have managed to control the epidemic. After this point, the interest turned to a n obvious question – whether antiretroviral drugs could be used to prevent infections? This led to a major success in preventing mother-to-child transmission of the virus, which now needs to be scale up to reach more HIV-infected pregnant women in poor settings. The main progress this year has been reported by the New England Journal of Medicine: the HIV Prevention Trials Network (HPTN) 052 study has provided definitive proof that antiretroviral treatment reduces the rate of sexual transmission of HIV-1. The international trial enrolled nearly 1800 discordant couples, and the HIV-1–positive partners were randomly assigned to receive early or delayed antiretroviral therapy (when the CD4 count dropped below 250 cells per cubic millimetre or an HIV-1–related event occurred). The study showed that antiretroviral treatment of the positive partner reduced the rate of transmission to the negative partner by more than a staggering 95% Also, immediate therapy slowed disease progression in the HIV-1–infected index patient as compared with delayed therapy, with a reduction of nearly 40% and with extrapulmonary tuberculosis dominating the clinical events and driving the between-group difference.

### A real progress being made by seekers of another Earth

Among astronomers, the past year will certainly be remembered for the real progress being made in search for Earth-like planets in the Universe. This has been one of the main goals of NASA's Kepler telescope in recent years, which has a capacity of planet-spotting in distant Universe – a relatively recent opportunity for astronomers. Writing for Nature in December this year, the scientists explained that Kepler spotted Earth sized and a Venus-sized planets in the same star system, which is about 1000 light years away from us. Both planets seem to orbit far too close to their parent star to be habitable, though. Francois Fressin, an astronomer at the Harvard-Smithsonian Center for Astrophysics in Cambridge, Massachusetts, who was the lead author of the paper, declared this discovery to be a “beginning of an era”, adding that we’ll soon be able to detect these kind of planets around other stars and at other distances. Kepler spots planets when they cross the face of their host stars and dim the light transmitted by the stars. This detection is very difficult when planets are as small as Earth, and researchers need to record many such transits to gain confidence in their discovery.

### Antibiotic resistance predates modern antibiotic treatment by at least 30 000 years

Intuitively, many researchers assumed that bacteria began to develop antibiotic resistance exclusively as a response to antibiotic treatment which has been widely used by humans over the past several decades. However, other scientists have been suspecting for some time that bacteria have been carrying antibiotic-resistance genes since ancient times. Most antibiotics that are currently in use are developed from toxic molecules which are being produced naturally by bacteria or fungi. This has led microbiologists to suspect that genes which confer resistance to these toxic molecules should constitute a natural part of many genomes of microorganisms. Sporadic reports claimed to have found resistance genes in bacterial samples taken from ancient sources, such as permafrost, but most studies of ancient DNA are plagues by concern that the samples may have been contaminated with modern DNA. But Gerry Wright, scientific director of the Michael G. DeGroote Institute for Infectious Disease Research, and Hendrik Poinar, McMaster University evolutionary geneticist, were able to develop methods to isolate small stretches of ancient DNA from microbes frozen in 30 000-year-old permafrost soil from the Yukon Territories. Publishing in Nature earlier this year, they discovered that antibiotic resistant genes existed beside genes that encoded DNA for ancient life, such as mammoths, horse and bison, as well as plants only found in that locality during the last interglacial period in the Pleistocene era.

